# Supplementary figures and images for: Smart Reaction Templating: A Graph-Based Method for Automated Molecular Dynamics Input Generation
Source: J Chem Inf Model. 2025 Jun 6;65(12):6038–47. doi: 10.1021/acs.jcim.5c00445 (PMC12199293; doi:10.1021/acs.jcim.5c00445)

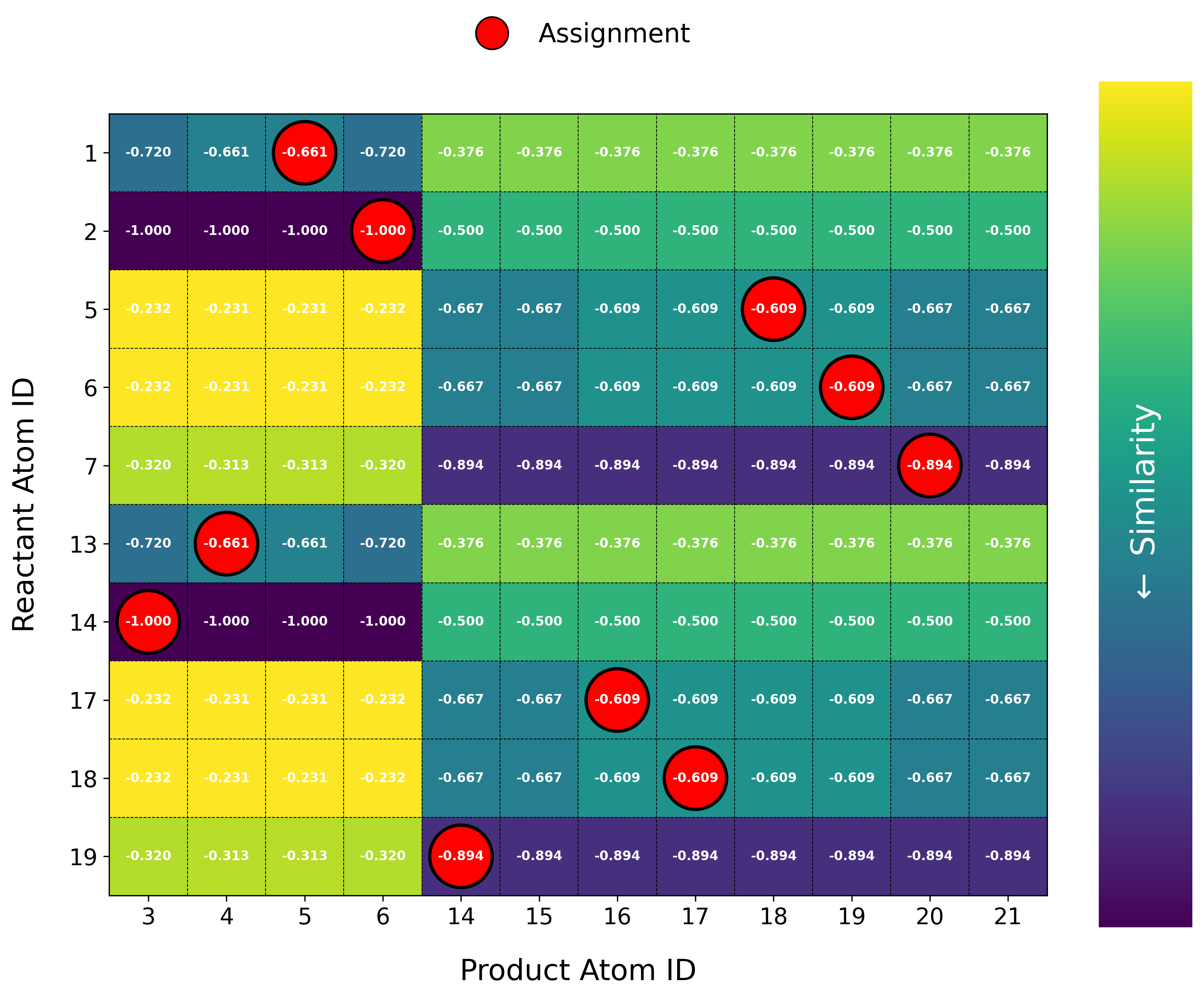

Supplement: Supplementary file 1 [file ci5c00445_si_001.zip › chain_polymerization/system/map/octane_swap_assignment.png]

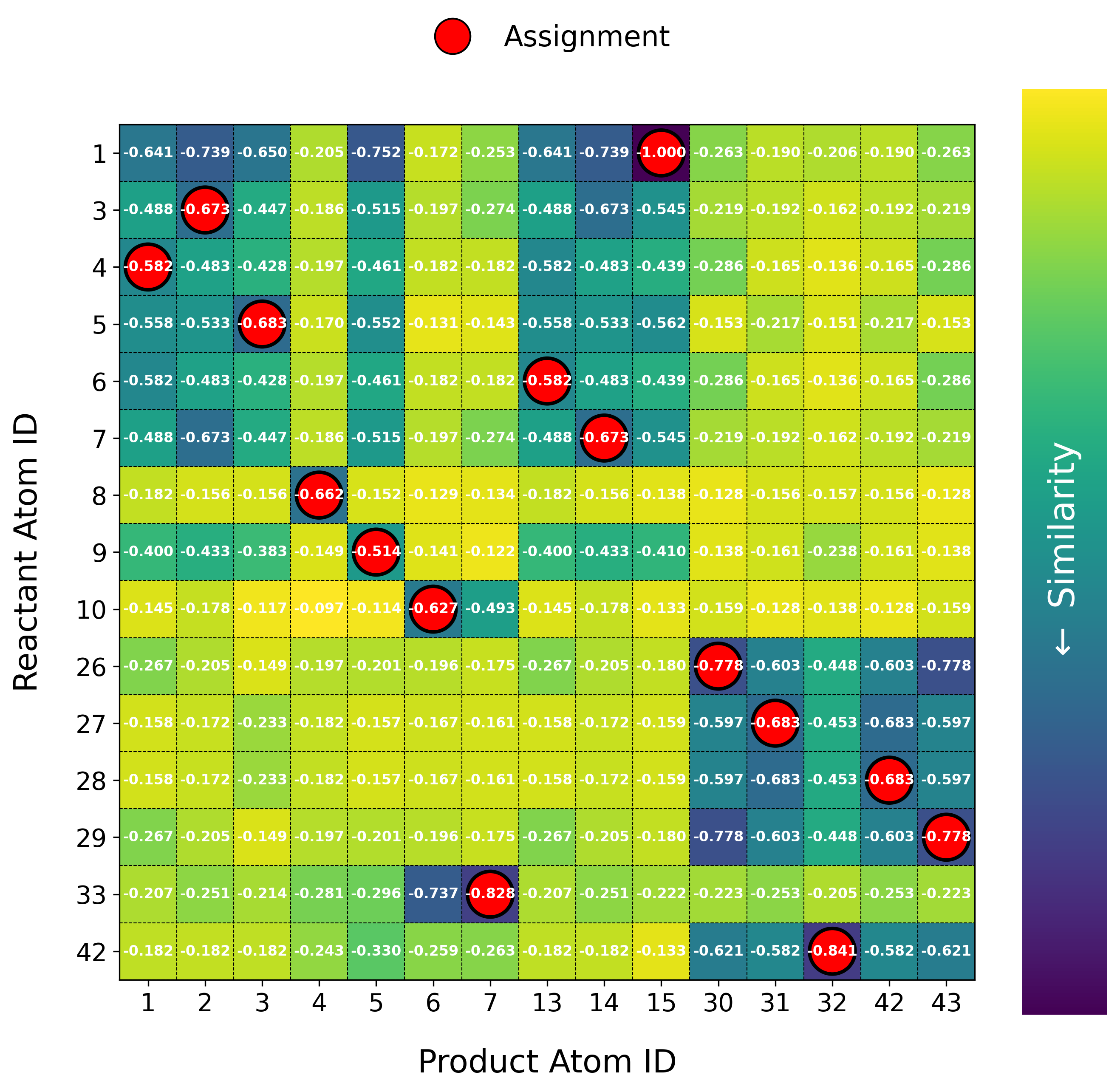

Supplement: Supplementary file 1 [file ci5c00445_si_001.zip › polyaddition/system/map/urethane_path_assignment.png]

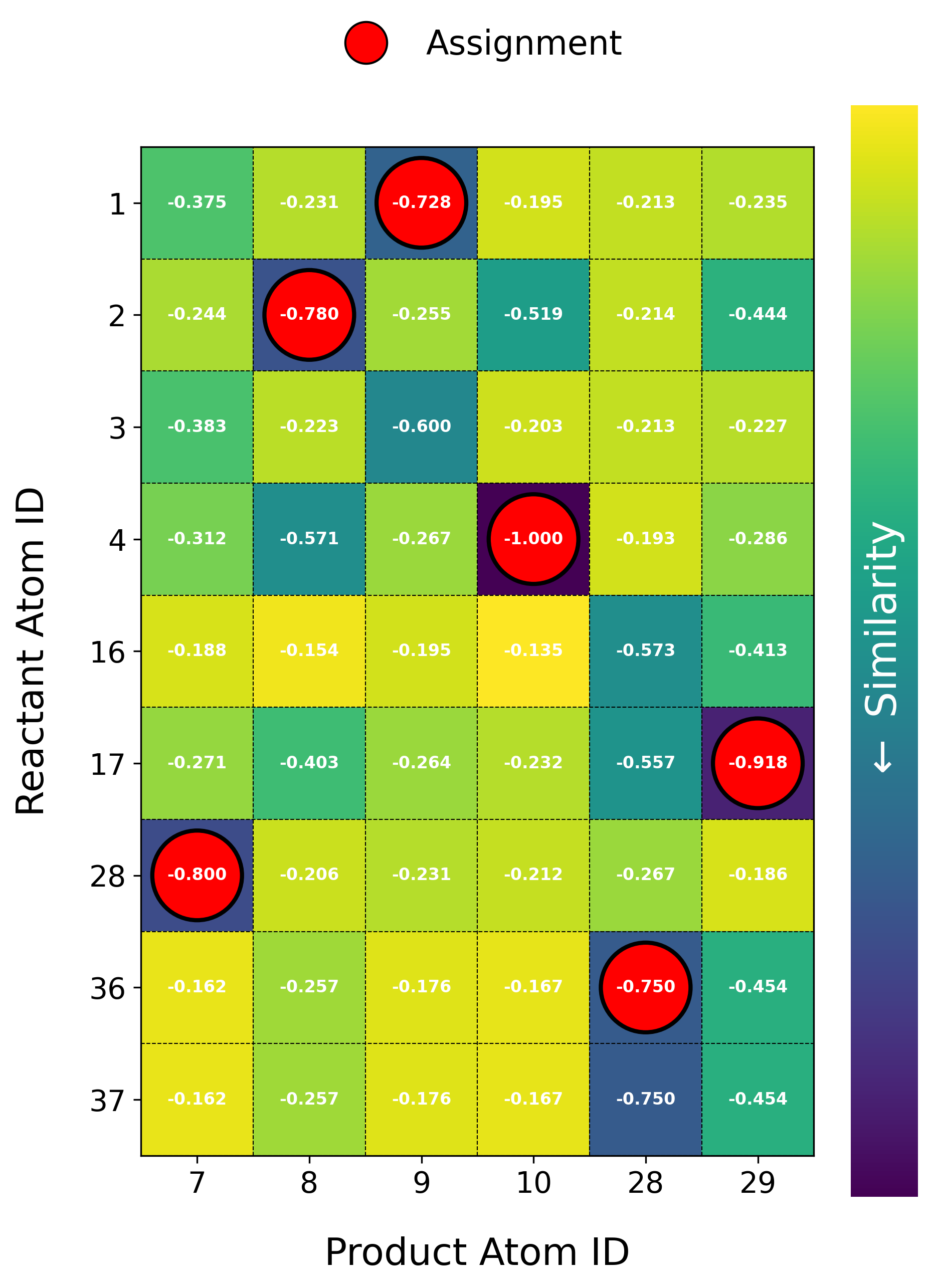

Supplement: Supplementary file 1 [file ci5c00445_si_001.zip › polycondensation/system/map/link1_path_assignment.png]

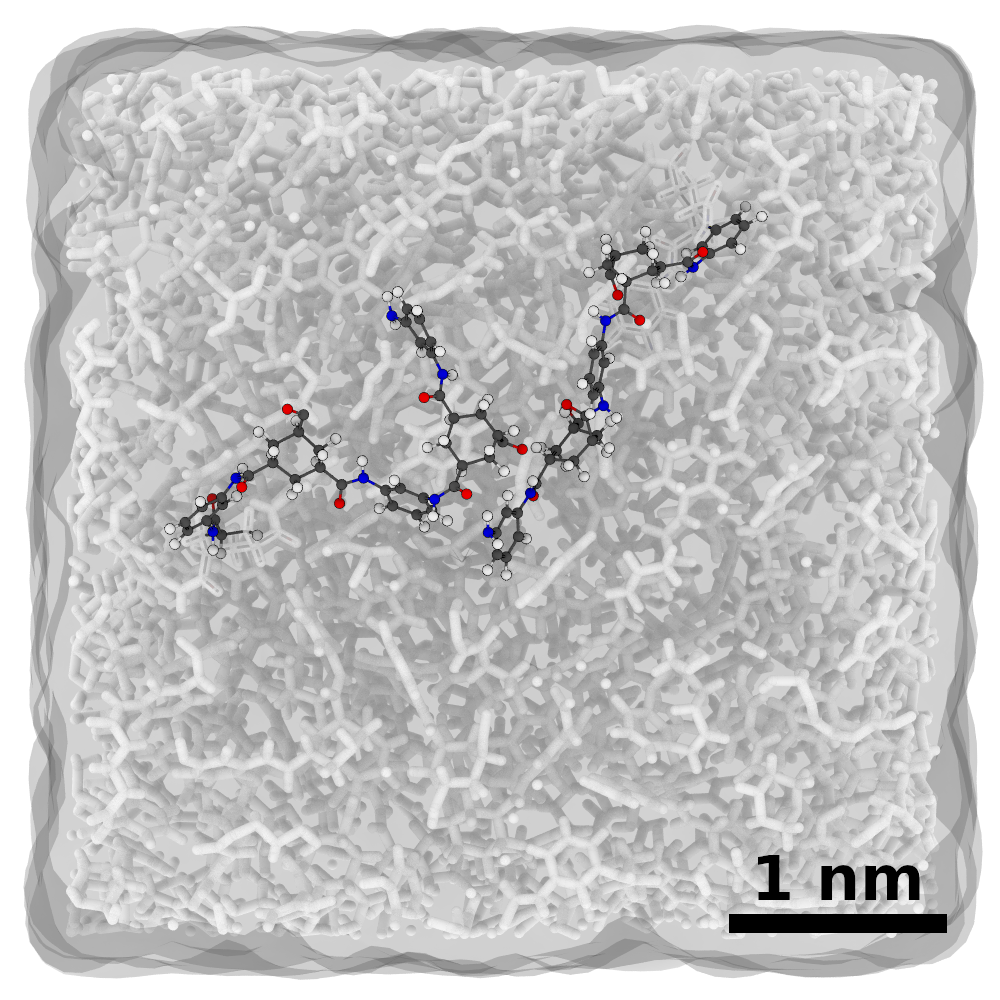

Supplement: Supplementary file 1 [file ci5c00445_si_001.zip › polycondensation/system/system_marked.png]

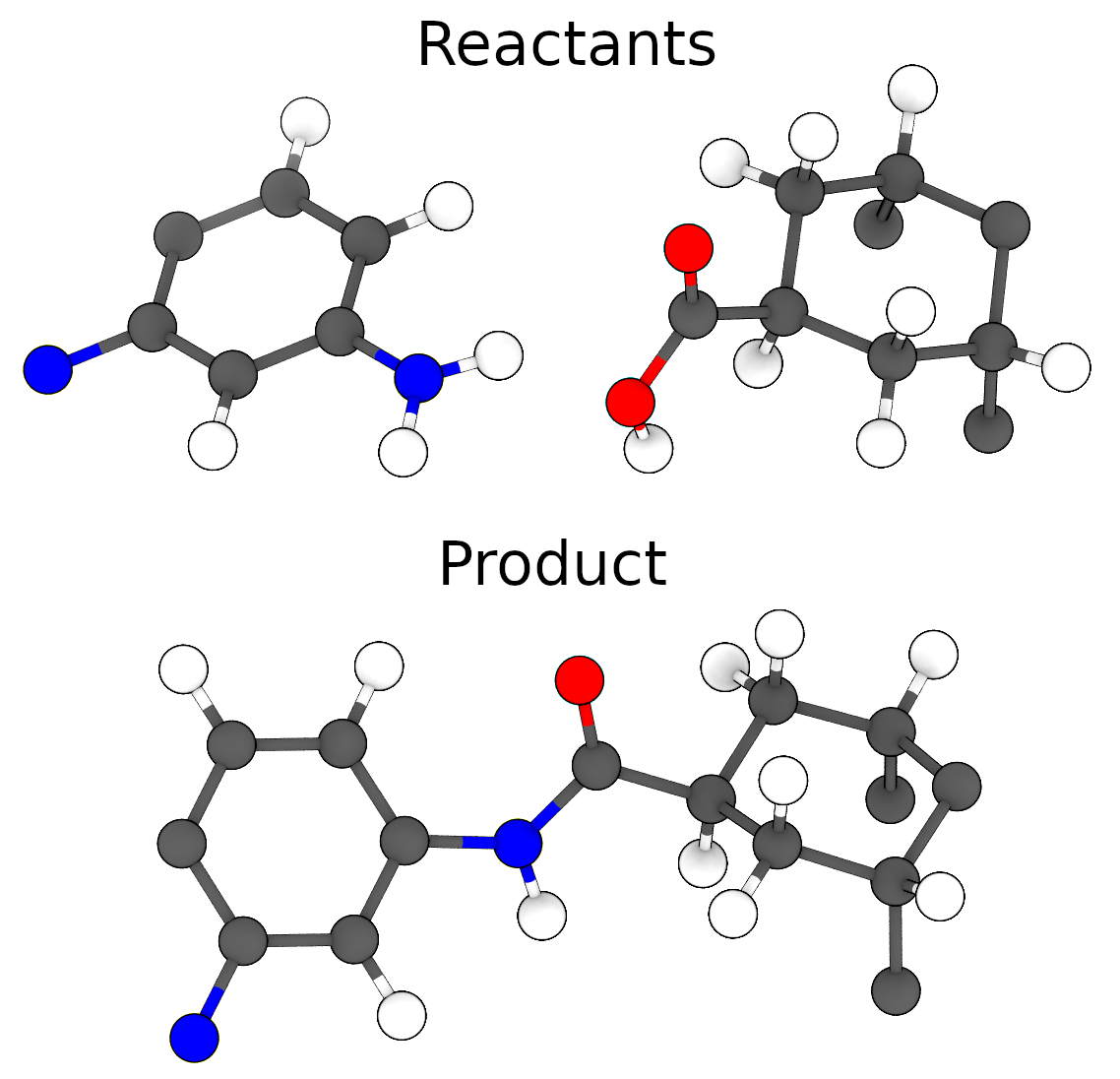

Supplement: Supplementary file 1 [file ci5c00445_si_001.zip › polycondensation/system/templates.png]

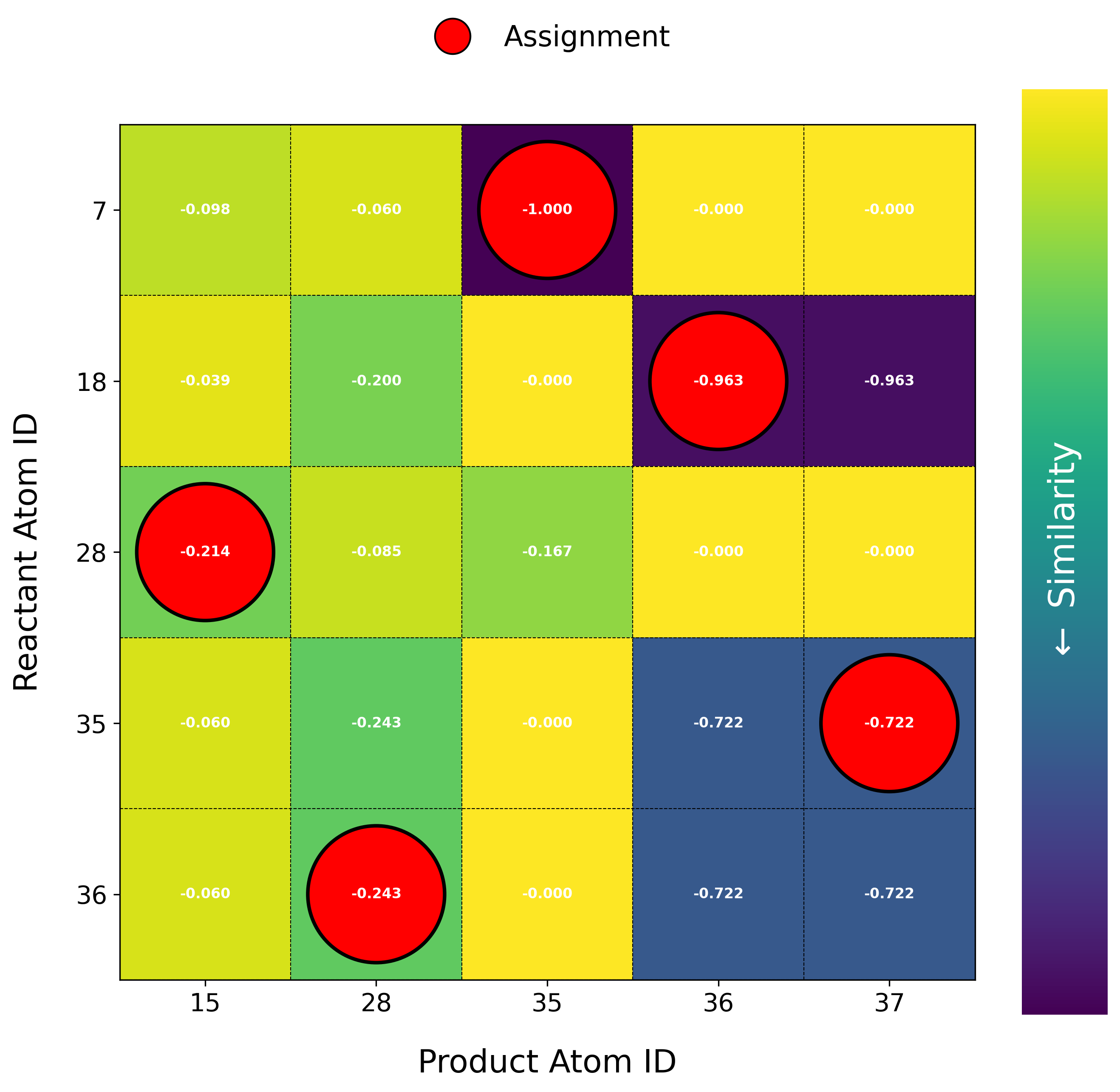

Supplement: Supplementary file 1 [file ci5c00445_si_001.zip › polycondensation_water/system/map/link1+spce_path_assignment.png]
